# Ethics and Technology in Nursing Education: Rethinking Artificial Intelligence

**DOI:** 10.17533/udea.iee.v43n3e14

**Published:** 2025-11-06

**Authors:** Palmira Canales-Morales

**Affiliations:** 1 Nurse, Ph.D Candidate. Professor, Universidad Santo Tomás, Sede Santiago, Chile. Email: palmiracanalesmo@santotomas.cl. Corresponding autor. https://orcid.org/0000-0002-4649-6780 Universidad Santo Tomás Universidad Santo Tomás Sede Santiago Chile palmiracanalesmo@santotomas.cl

**Keywords:** artificial intelligence, ethics, nursing, education, nursing, health equity., inteligencia artificial, ética en enfermería, educación en enfermería, equidad en salud., inteligência artificial, ética em enfermagem, educação em enfermagem, equidade em saúde.

## Abstract

This essay aims to critically reflect on the ethical challenges posed by the incorporation of artificial intelligence (AI) in the education of nursing professionals. Based on an argumentative review of recent scientific literature, three key dimensions are analyzed: personalized learning as an opportunity to promote inclusion and academic autonomy; the potential of AI to reduce educational gaps through public policies that guarantee digital equity; and the strengthening of clinical and ethical judgment through AI-assisted simulations in educational environments. Likewise, the risks associated with its implementation are addressed, such as algorithmic surveillance, the mechanization of moral reasoning, and technological exclusion in contexts with low digital literacy. It is concluded that the real challenge does not lie in the technology itself, but in how it is designed, regulated, and pedagogically integrated in terms of care, dignity, and critical thinking. Finally, the need to advance research that guides regulatory frameworks and educational proposals to ensure an ethical, inclusive, and contextualized integration of AI in nursing education is suggested.

## Resumen

Este ensayo tiene como objetivo reflexionar críticamente sobre los desafíos éticos que plantea la incorporación de la inteligencia artificial (IA) en la formación de profesionales de enfermería. A partir de una revisión argumentativa de literatura científica reciente, se analizan tres dimensiones clave: la personalización del aprendizaje como oportunidad para favorecer la inclusión y la autonomía académica; el potencial de la IA para reducir brechas educativas mediante políticas públicas que garanticen equidad digital; y el fortalecimiento del juicio clínico y ético a través de simulaciones asistidas por IA en entornos formativos. Asimismo, se abordan los riesgos asociados a su implementación, como la vigilancia algorítmica, la mecanización del razonamiento moral y la exclusión tecnológica en contextos con baja alfabetización digital. Se concluye que el verdadero desafío no radica en la tecnología en sí, sino en cómo se diseña, regula e integra pedagógicamente en función del cuidado, la dignidad y el pensamiento crítico. Finalmente, se sugiere la necesidad de avanzar en investigaciones que orienten marcos normativos y propuestas educativas que aseguren una integración ética, inclusiva y contextualizada de la IA en la formación en enfermería.

## Resumo

Este ensaio tem como objetivo refletir criticamente sobre os desafios éticos impostos pela incorporação da inteligência artificial (IA) na formação de profissionais de enfermagem. A partir de uma revisão argumentativa da literatura científica recente, são analisados ​​três aspectos-chave: a personalização da aprendizagem como oportunidade para promover a inclusão e a autonomia acadêmica; o potencial da IA ​​para reduzir lacunas educacionais por meio de políticas públicas que garantam a equidade digital; e o fortalecimento do julgamento clínico e ético por meio de simulações assistidas por IA em ambientes de formação. Também são abordados os riscos associados à sua implementação, como a vigilância algorítmica, a mecanização do raciocínio moral e a exclusão tecnológica em contextos de baixa alfabetização digital. Conclui-se que o verdadeiro desafio não está na tecnologia em si, mas em como ela é projetada, regulada e integrada pedagogicamente com base no cuidado, na dignidade e no pensamento crítico. Por fim, sugere-se a necessidade de avançar em pesquisas que orientem marcos regulatórios e propostas educacionais que garantam a integração ética, inclusiva e contextualizada da IA ​​na educação em enfermagem.

## Introduction

Artificial intelligence (AI), since it was coined in 1956 at the Dartmouth Conference by John McCarthy, has gone through a long path of technological evolution. In 2022, this trajectory took a transcendental turn with the emergence of generative artificial intelligence (GenAI), marking a before and after in multiple fields, including higher education.^1^ This advance, although fascinating, has exceeded the capacity of societies to regulate its impacts. While legitimate ethical concerns persist, the potential of GenAI to transform educational practices and strengthen the preparation of future health professionals is undeniable.^2^ In the health field, AI promises to optimize processes, improve diagnoses, and personalize learning experiences. However, its adoption faces important ethical and practical barriers, such as trust in systems, algorithmic transparency, and effective integration into complex clinical workflows.^3^ In the specific field of nursing, technologies such as chatbots or AI-enhanced simulators have positioned themselves as promising tools to enrich training: they allow for more adaptive, accessible, and motivating learning. Even so, their incorporation is not free of ethical tensions, especially in a discipline where care, empathy, and social responsibility constitute fundamental pillars of the educational act.^4^

As these technologies are integrated into the classroom, it becomes essential that teachers and students develop the competencies to use them with critical and ethical sense. Issues such as privacy, algorithmic biases, student autonomy, and the use of sensitive data require not only deep digital literacy, but also regulatory and pedagogical frameworks that guide their implementation. In this sense, it has been proposed to advance in three strategic areas: the construction of solid ethical frameworks, the promotion of interdisciplinary collaboration, and investment in continuous training for all the actors involved.^5^ These strategies seek to ensure that AI does not deepen existing inequalities, but rather becomes a tool at the service of the common good, sustained in values such as equity, inclusion, and safety. In this context, GenAI cannot be understood solely as a technical leap, but as a transformation that directly questions the ethical and pedagogical foundations of teaching, especially in a discipline such as nursing, where technical, humanistic, and ethical knowledge converge. As various authors warn, the ethical dilemmas that emerge with AI range from privacy and individual autonomy to broader issues of social justice, environmental sustainability, and civil rights.^6^ When these dilemmas are transferred to the educational field, they directly affect the way future generations of health professionals are trained.

The expansion of automated systems, personalization algorithms, and adaptive platforms poses concrete risks: the dehumanization of pedagogical relationships, the standardization of learning, and the reproduction of social and digital inequalities. Added to this is the concern about a possible loss of autonomy both for students and teachers, when educational decisions begin to be delegated to systems that are opaque and difficult to audit.^6^ In view of this panorama, the present essay adopts a critical, reflective, and purposeful stance regarding the role of artificial intelligence in the training of nurses. Based on the analysis of recent scientific evidence, it examines the risks and opportunities that this technology represents for health education, with the aim of proposing guidelines that allow for its ethical, contextualized, and humanized integration. The thesis that guides this work is that AI can become a valuable ally in educational processes, as long as its implementation is guided by ethical, pedagogical, and social principles oriented towards the care of human dignity, educational justice, and critical thinking.

From this perspective, the essay is structured around three central arguments. First, the potential of AI to personalize learning is addressed, adapting to the pace and needs of each student, which could help reduce academic dropout and strengthen autonomy. Second, its capacity to promote greater educational equity is explored, provided that its implementation is accompanied by inclusive public policies and strategies to reduce the digital divide. Finally, the role of AI in strengthening clinical and ethical judgment through formative simulations is analyzed, without losing sight of the risks of mechanization of moral reasoning. In short, this essay seeks to open an academic and ethical debate on how to critically inhabit technology in the formative processes of nursing. It is not about rejecting AI nor assuming it with naïve enthusiasm, but about collectively thinking about how to integrate it without giving up what is essential: the sensitivity, the ethics, and the human encounter that give meaning to care.

### Personalization of Learning: A Pedagogical Opportunity with Ethical Risks

The integration of artificial intelligence (AI) into nursing education presents itself as a profoundly transformative pedagogical strategy. Its ability to personalize learning, adapt content to individual needs, and provide immediate feedback offers unique opportunities to optimize the educational process.^7^^,^^8^ Through real-time data analysis, AI-based systems can adjust the pace, formats, and teaching strategies, responding to students’ diverse skill levels and learning styles.^8^ In contexts where student groups are large and heterogeneous, this technology acts as a strategic ally of faculty, allowing them to address differences that would otherwise go unattended. The aim is not to replace teachers but to expand and strengthen their capacity for accompaniment, especially when faced with limitations of time or resources. This type of support can have a direct impact on students’ self-esteem, motivation, and autonomy, by offering a fairer and more inclusive learning experience.^9^ Furthermore, the personalization that AI offers helps address one of the major challenges of higher education in Latin America: student attrition. Several studies have shown that low academic performance, curricular misalignment, lack of effective feedback, and limited faculty support are determining factors in university dropout.^10^^,^^11^ In health-related programs, these risks are accentuated during the first semesters, when adapting to the pace of university life is crucial.^12^

In this scenario, AI can provide concrete solutions: by offering adaptive practices, simulating clinical scenarios, identifying learning gaps in a timely manner, and providing personalized support, academic trajectories are strengthened, and student retention is improved. For example, intelligent tools can identify students with difficulties in pharmacology and generate additional exercises to reinforce their learning something difficult to achieve with traditional methods in overcrowded classrooms. In nursing, where the acquisition of clinical, cognitive, and ethical competencies requires constant practice, aI-powered simulated environment offer a safe and flexible alternative. These platforms allow students to repeat procedures, make mistakes without real-world consequences, and receive immediate feedback.^13^ This democratizes access to learning opportunities that, in real clinical settings, are often distributed unequally. From an ethical perspective, this personalization represents a concrete way of applying the principle of justice in education, by providing equal training opportunities, reducing access gaps, and enabling each student to develop their potential. Likewise, the principle of beneficence is reflected in the commitment to use technologies that favor students’ academic and integral well-being.

### Towards Ethical Surveillance and Strengthened Autonomy

Concerns about algorithmic surveillance and the loss of autonomy are legitimate and must be taken seriously. However, it is important to recognize that the risks associated with the use of artificial intelligence (AI) in education do not derive from the technology itself, but from the way it is designed, managed, and implemented. Therefore, the challenge is not merely technical, but also ethical and political: the focus should be on promoting critical governance of AI, articulating clear regulatory frameworks, processes of informed consent, algorithmic transparency, and the active participation of students in the decisions that affect them. In this regard, it has been pointed out that AI-supported educational systems should empower students, allowing them to understand, audit, and even intervene in automated recommendations.^18^ This transparency not only protects fundamental rights but also strengthens their autonomy, positioning them as active subjects in their learning process. To achieve this, it is essential to incorporate ethical training and digital literacy from the early stages of the educational system, as recommended by the United Nations Educational, Scientific and Cultural Organization.^7^

Beyond understanding autonomy as a merely individualistic ideal, it can also be conceived from a relational perspective: if implemented critically and reflectively, AI can act as an extension of the self-learning process, enabling each student to make more informed decisions about their personal and professional development.^9^ In this context, the role of teachers does not disappear but is transformed. By being freed from repetitive or administrative tasks, new spaces open up to strengthen critical mentoring, emotional support, and the teaching of ethical and professional values.^2^ It is also imperative that states assume an active and regulatory role regarding these technologies. The misuse of educational data undermines fundamental rights such as privacy, informational self-determination, and equity. Regulatory models such as the General Data Protection Regulation (GDPR) in Europe, the Family Educational Rights and Privacy Act (FERPA) in the United States, or emerging policies in Asian countries can serve as references to guarantee the protection of students’ data and establish minimum standards of digital ethics in the educational field.^19^ In sum, AI can and must contribute to more inclusive, personalized, and effective education, provided that its integration is guided by solid ethical principles, robust regulatory frameworks, and critical human oversight. The point is not to renounce technology, but to inhabit it consciously, safeguarding the dignity, privacy, and autonomy of learners

### Artificial Intelligence as a Promoter of Educational Equity

Educational equity is an ethical principle that seeks to ensure that all students-regardless of their social, economic, or cultural background-have access to quality education. Unlike equality, which implies offering the same to everyone, equity acknowledges structural inequalities and proposes differentiated support to provide each person with real opportunities for learning and development.^19^ Among its key dimensions are the fair distribution of resources, the strengthening of teaching capacities, the design of inclusive pedagogical practices, and the constant monitoring of gaps with the aim of proactively reducing them. AI, when implemented through a critical and pedagogically oriented lens, can significantly contribute to these purposes. AI-based tools allow for the adaptation of content, the delivery of personalized feedback, the generation of accessible materials, and the early detection of academic difficulties, thereby facilitating more effective support that is sensitive to diversity.^20^^,^^21^ From this perspective, AI positions itself as a tool with great potential to foster inclusive educational environments. Technologies such as voice recognition, screen readers, automatic transcription, and subtitles help eliminate historical barriers faced by students with disabilities. Likewise, early warning systems that analyze academic data can identify risks of dropout or academic delay, allowing for timely interventions, especially during the first semesters of university studies.^21^ AI also promotes methodological diversification by tailoring teaching to different cognitive styles, learning paces, and educational trajectories. This personalization is particularly valuable for vulnerable populations, such as students who work, who perform caregiving duties, or who belong to cultural or linguistic minorities. In this sense, educational equity is not limited only to access, but also to the relevance, depth, and quality of learning.

Nevertheless, for this personalization to be ethical, it is essential to consider the risks associated with algorithmic bias. Several studies have demonstrated that AI systems can reproduce and even amplify preexisting prejudices, depending on how they were trained. These biases may arise from incomplete data, erroneous assumptions by developers, or non-representative datasets.^22^^,^^23^ In education, this could translate into unfair decisions that negatively affect academic trajectories, reinforcing stereotypes of gender, race, or social class. As an illustrative example, a generative AI system was asked how it perceives university students, particularly those studying nursing.^24^ The responses revealed troubling stereotypes: the idealization of young students, the invisibilization of non-linear educational paths, the assumption of homogeneous access to technology, the undervaluing of the emotional dimensions of learning, and even gender and professional subordination biases. Although generated by an automated system, these responses reflect social imaginaries still in force, which risk being reinforced if not critically addressed. All of this shows that AI is not a neutral technology. When integrated into teaching processes-especially in contexts such as nursing, where technical, ethical, and humanistic knowledge converge-it becomes imperative to question what discourses it reinforces, what experiences it excludes, and what criteria it reproduces in decision-making. If these logics are not critically reviewed, we could end up perpetuating the very prejudices we seek to transform.

### Deepening of Structural Digital Divides

Despite its inclusive potential, the implementation of artificial intelligence (AI) can also exacerbate existing inequalities if structural gaps are not critically addressed. It has been noted that access to these technologies varies enormously across countries, regions, and educational communities.^7^ While institutions in high-income contexts often have stable connectivity, technological resources, and political support, many areas of the Global South still face basic deficiencies such as access to devices, continuous electricity, or even elementary digital literacy. These digital divides are not limited solely to material access to the internet or technological tools; they also include the ability to use them critically, to adapt them to local realities, and to integrate them pedagogically into learning processes.^20^ In this scenario, a rapid deployment of AI without accompanying public policies-such as infrastructure development, teacher training, technical support, and sustained funding-could generate new forms of exclusion. Some students may not understand how these tools work, may lack adequate guidance, or may only have access to outdated versions of platforms, thereby deepening educational inequality.

Another significant risk is the imposition of pedagogical and epistemological models disconnected from local contexts. Many AI systems are developed in urban, technologically advanced, and culturally homogeneous environments, where English predominates and knowledge is shaped by standardized logics. By failing to incorporate the cultural, linguistic, and community richness of rural areas or Indigenous peoples, such tools risk reinforcing technological dependence, invisibilizing local knowledge, and reproducing colonial hierarchies of knowledge.^21^ In short, digital equity is not achieved solely by providing more technology, but through intersectoral policies that recognize the multiple dimensions of inequality and ensure fair conditions for access, appropriation, and participation. Otherwise, AI could become not a bridge, but a new boundary that deepens the very inequalities it aims to overcome.

### Digital Equity Requires Political Will, Not Technological Abandonment

The risk of deepening inequalities should not lead to abandoning the use of artificial intelligence (AI) in education. On the contrary, it constitutes an ethical call to transform it into an ally for educational justice. The dilemma does not lie in whether to use technology, but in how, for what, and for whom it is implemented.^7^^,^^25^ Achieving true digital equity requires more than access to infrastructure: it demands sustained investment in connectivity, continuous teacher training, the production of culturally relevant content, the development of ethical frameworks, and the active participation of educational communities. Equity is not achieved merely by providing more devices, but by fostering inclusive pedagogies, empowering teachers, and preparing students capable of critically understanding the digital world they inhabit.^19^ Some countries have shown that an ethical and equitable integration of AI is possible.

Experiences developed in contexts such as Finland, Singapore, or Estonia demonstrate that, with coordinated public policies, teacher training programs, and continuous evaluation mechanisms, it is possible to align technological innovation with the principles of equity, inclusion, and democratic participation.^19^ These examples show that AI is not exclusionary by nature; it becomes exclusionary when it is deployed without context, without participation, and without clear ethical criteria. Ultimately, the issue is not to idealize technology nor to assume it as a panacea. It is about committing pedagogically and politically to a more just education, in which AI is not a privilege reserved for a few, but a right built collectively. Digital equity is possible, but it requires courageous decisions, institutional will, and an ethics radically committed to the dignity of all students.

### Strengthening Clinical and Ethical Judgment through Simulated Environments

Clinical judgment constitutes an essential competency in the training of nursing professionals, as it enables prudent and well-founded decisions in complex care scenarios. This judgment is not limited to technical mastery: it involves ethical reasoning, moral sensitivity, and the ability to act with discernment in the face of human suffering.^2^^,^^17^ However, several studies have indicated that this competency is not always adequately developed during professional training, revealing the need for a profound renewal of the pedagogical strategies employed in educational processes.^5^^,^^18^ In this context, clinical simulation-especially high-fidelity simulation-has become a key strategy for strengthening clinical and ethical judgment in nursing.^9^ With the incorporation of artificial intelligence (AI), these educational experiences acquire a new dimension. Advanced platforms make it possible to recreate complex clinical scenarios, with adaptive and immediate feedback that guides students in their decision-making. These simulations can include ethical dilemmas such as care prioritization, informed consent, or patient confidentiality, integrating technical and moral components in a single educational experience.

Moreover, these tools allow the representation of emotions, uncertainties, and tensions characteristic of real clinical settings. It has been documented that AI-assisted simulation environments not only strengthen clinical reasoning but also promote skills such as emotional self-regulation, empathy, and interprofessional communication.^4^^,^^5^^,^^9^ These abilities are fundamental in nursing practice, where the human dimension of care cannot be replaced by any algorithm. From a phenomenological perspective, it has been proposed that clinical judgment is constructed in contexts of uncertainty, where the professional must respond to the suffering of others with practical wisdom based on experience and situated reflection.^17^ In this sense, AI-enhanced simulations can place students in ethically challenging scenarios, where they must confront decisions without unique answers, deliberate responsibly, and reflect on the deeper meaning of their actions. Thus, technology does not replace moral sensitivity but cultivates it through meaningful educational experiences.

Nevertheless, critical voices also warn about the risks of an education excessively mediated by intelligent technologies. It has been argued that the automation of simulations could create the false impression that there is only one correct answer, thereby inhibiting ethical judgment and limiting divergent thinking.^4^ Likewise, excessive dependence on digital platforms could weaken clinical intuition and the ability to interpret context-elements that are indispensable in nursing practice.^18^ These warnings call for deep reflection: How can we integrate technology without impoverishing the ethical dimension of care? How can we train professionals who are technically competent but also ethically sensitive and empathetic? The key does not lie in choosing between technology or humanism, but in articulating both with pedagogical intent, critical openness, and commitment to the dignity of others.

### Clinical Judgment Is Strengthened in Ethically Designed Environments

Clinical judgment cannot be reduced to a technical skill, nor does it develop spontaneously. It is a competency cultivated in carefully designed pedagogical environments that promote critical reflection, ethical deliberation, and meaningful learning. Far from replacing these processes, artificial intelligence (AI) can enhance them, provided its integration is guided by a humanized educational intent and accompanied by teachers committed to care.^2^ Evidence has shown that when AI is incorporated into clinical simulation environments in a critical and deliberative manner, it can strengthen students’ ability to reflect on ethical dilemmas and make complex decisions in simulated contexts.^4^^,^^9^ In these scenarios, AI does not provide automatic answers or impose closed criteria; instead, it poses challenges that invite students to think, argue, and assume responsibility for their own judgment.

International recommendations on the ethical use of AI in education emphasize the need for these technologies to promote human dignity, inclusion, and critical thinking.^7^ Simulating ethical dilemmas with AI does not mean automating moral responses, but recreating experiences that challenge students, confront them with possible alternatives, and invite them to assume responsibility for their decisions. When these tools are combined with faculty guidance and spaces for collective reflection, they become allies of clinical judgment, not threats to it. The key lies in pedagogical design. It is not about technology deciding for students, but about creating experiences that stimulate their ethical discernment. It is not about replacing deliberation, but provoking it. Because in nursing, deciding is also an act of care, and caring always requires an ethical, situated, and profoundly human perspective.^17^^,^^18^

## Conclusions

This essay has maintained the thesis that artificial intelligence (AI), when incorporated into nursing education from an ethical, inclusive, and pedagogically intentional perspective, can become a powerful tool to enrich learning without compromising the humanized dimension that characterizes care. Far from a technophobic or technocratic stance, a critical and situated perspective has been proposed, one that makes it possible to engage with technology without abandoning the principles that give meaning to health education. Throughout the text, three main arguments were developed, each accompanied by its respective counterargument and refutation, in order to explore both the possibilities and the tensions that arise when introducing intelligent technologies into educational spaces in health.

First, it was argued that AI has enormous potential to personalize learning, adapting to the individual needs of students in contexts marked by heterogeneity and structural limitations. This adaptive capacity fosters autonomy, motivation, and academic performance. However, warnings were raised about the risks of algorithmic surveillance and loss of privacy, with transparent regulatory frameworks and student participation mechanisms proposed as urgent responses to ensure ethical and informed accompaniment.^9^^,^^14^

Second, it was maintained that AI could become an ally in reducing historical educational gaps, provided it is embedded in inclusive public policies, continuous teacher training, and strategies for critical digital literacy. Its potential to generate accessibility, identify specific needs, and diversify educational resources is especially relevant in highly vulnerable contexts. Nevertheless, it was also recognized that, without state investment and contextualized design, technology risks deepening structural and epistemic inequalities. Therefore, the ethical requirement of conceptualizing AI from its origin with criteria of justice, equity, and cultural relevance was reaffirmed.^9^^,^^19^^,^^20^

The third argument addressed the contribution of AI to strengthening students’ clinical and ethical judgment, particularly through pedagogically designed clinical simulations. These tools make it possible to recreate complex care scenarios, provide immediate feedback, and promote prudent, empathetic, and safe decision-making. Faced with fears that such technologies could mechanize moral reasoning, an alternative perspective was proposed: AI does not replace ethical deliberation, but can enhance it when integrated into pedagogical environments guided by teachers trained in the ethics of care. From a phenomenological approach, it was argued that these formative experiences can consolidate the professional identity of caregivers, by placing them in contexts of uncertainty, responsibility, and compassion.^2^^,^^5^^,^^17^

Taken together, these three arguments allow us to affirm that AI does not constitute an intrinsic threat to nursing education. Its impact will depend on the ethical and political sense that guides its incorporation. The counterarguments raised-algorithmic surveillance, digital exclusion, and mechanization of judgment-should not be interpreted as reasons to reject technology, but as necessary warnings that reinforce the urgency of designing critical governance, developing inclusive policies, and promoting context-sensitive pedagogies. As various studies warn, the ethics of AI in education is today an emerging field that must be addressed with urgency. It is not enough to teach how to use these tools; it is imperative to train professionals capable of questioning them, transforming them, and orienting them toward the common good.^6^ Artificial intelligence should not be imposed as a neutral promise, but as a tool that must be at the service of life, justice, and care.

Finally, this essay invites us to rethink the meaning of the educational act in times of automation. Training in nursing is not simply about transmitting techniques or integrating devices, but about cultivating critical thinking, empathetic sensitivity, and an ethic of care capable of resisting the logic of efficiency and keeping the human dimension at the center of learning. On this path, artificial intelligence should not displace care, but rather reinforce its depth, its complexity, and its dignity.

## References

[1] Mondal H (2025). Ethical engagement with artificial intelligence in medical education. Advances in Physiology Education.

[2] Lane SH, Haley T, Brackney DE (2024). Tool or Tyrant: Guiding and Guarding Generative Artificial Intelligence Use in Nursing Education. Creative Nursing.

[3] Esmaeilzadeh P (2024). Challenges and strategies for wide-scale artificial intelligence (AI) deployment in healthcare practices: A perspective for healthcare organizations. Artificial Intelligence in Medicine.

[4] Tam W, Huynh T, Tang A, Luong S, Khatri Y, Zhou W (2023). Nursing education in the age of artificial intelligence powered Chatbots (AI-Chatbots): Are we ready yet?. Nurse Education Today.

[5] Srinivasan M, Venugopal A, Venkatesan L, Kumar R (2024). Navigating the Pedagogical Landscape: Exploring the Implications of AI and Chatbots in Nursing Education. JMIR Nursing.

[6] Huang C, Zhang Z, Mao B, Yao X (2023). An overview of artificial intelligence ethics. IEEE Transactions on Artificial Intelligence.

[7] UNESCO (2021). Recomendación sobre la ética de la inteligencia artificial [Internet].

[8] Yurt E (2024). Proceedings of the International Conference on Education.

[9] Sidiropoulos D, Anagnostopoulos CN (2024). Applications, challenges and ethical issues of AI and ChatGPT in education. Ciencias de la Computación.

[10] Ramírez Heredia RC, Carcausto-Calla W (2024). Factors associated with student dropout in Latin American universities: Scoping review. Journal of Educational and Social Research.

[11] Pérez-Jaimes AK, Estrada-Reyes CU, Hurtado-Velmar KP (2025). Factores causales de la deserción escolar en alumnos de nivel superior. Revista de Investigación Latinoamericana en Competitividad Organizacional.

[12] Martínez Reinoso AR, Laínez Tomalá AD, Bravo López JA, Cevallos Mendoza LA, Vera Delgado FV (2024). Factores asociados a la deserción estudiantil en los primeros años de carreras de salud: una revisión sistemática de intervenciones y estrategias de retención. Revista Social Fronteriza.

[13] American Nurses Association (2022). The ethical use of artificial intelligence in nursing practice [Internet].

[14] Oye E, Edwin F, Owen J (2024). Ethical considerations in AI-driven education. ResearchGate Preprint.

[15] Williamson B (2019). Policy networks, performance metrics and platform markets: Charting the expanding data infrastructure of higher education. British Journal of Educational Technology.

[16] Chinta SV, Wang Z, Yin Z, Hoang N, Gonzalez M, Le QT (2024). FairAIED: Navigating fairness, bias, and ethics in educational AI applications. AI and Ethics.

[17] Escobar-Castellanos B, Jara Concha P (2019). Filosofía de Patricia Benner, aplicación en la formación de enfermería: propuestas de estrategias de aprendizaje. Educación.

[18] Knopp MI, Warm EJ, Weber D, Kelleher M, Kinnear B, Schumacher DJ (2023). AI-Enabled Medical Education: Threads of change, promising futures, and risky realities across four potential future worlds. JMIR Medical Education.

[19] Vincent-Lancrin S, Van der Vlies R (2020). Trustworthy artificial intelligence (AI) in education.

[20] OECD (2023). Equity and inclusion in education [Internet].

[21] Melo-López VA, Basantes-Andrade A, Gudiño-Mejía CB, Hernández-Martínez E (2025). The impact of artificial intelligence on inclusive education: a systematic review. Education Sciences.

[22] Comas-Forjas R (2023). Proceedings of the 8th Virtual International Conference on Education, Innovation and ICT.

[23] Akgun S, Greenhow C (2022). Artificial intelligence in education: Addressing ethical challenges in K-12 settings. AI and Ethics.

[24] Sabzalieva E, Valentini A (2023). ChatGPT and artificial intelligence in higher education: quick start guide.

[25] Martínez V (2024). De qué hablamos cuando hablamos de inteligencia artificial [Internet].

